# Sudden Chest Pain Due to Esophageal Intramural Dissection: First Manifestation of Eosinophilic Esophagitis in a Teenager

**DOI:** 10.1097/PG9.0000000000000037

**Published:** 2020-12-09

**Authors:** Cigdem Arikan, Ozlem Yilmaz, Irem Yenidogan, Nuray Uslu Kizilkan, Fatih Aslan

**Affiliations:** From the * Pediatric Gastroenterology, Hepatology and Liver Transplantation, Koc University School of Medicine, Istanbul, Turkey; † Koc University Research Center for Translational Medicine (KUTTAM), Koc University School of Medicine, Istanbul, Turkey; ‡ Pediatric Allergy and Immunology, Koc University School of Medicine, Istanbul, Turkey; § Pediatrics, Koc University School of Medicine, Istanbul, Turkey; ¶ Gastroenterology, Koc University School of Medicine, Istanbul, Turkey.

## INTRODUCTION

Eosinophilic esophagitis (EoE) is a chronic disease with increasing prevalence initiated by food allergens (1). Mediated by T helper 2 inflammatory cascade, EoE precedes smooth muscle hypertrophy and fibrosis of the epithelium and subepithelium, consequently leading to stricture and functional motility abnormalities (2, 3). Young children mostly suffer from refusal of feeding and abdominal pain, whereas dysphagia and food impaction become more prominent at later childhood and adolescence. The chronic nature of EoE and the frequent diagnostic delay causes the uncontrolled remodeling which can be manifested with mucosal dissection (eosophageal intramural dissection [EID]) and perforation, but primary manifestation of EoE with EID is very rare (4–7). In this report, we presented a 14 years old boy with spontaneous EID who was suffered chest pain.

## CASE

**FIGURE 1.**
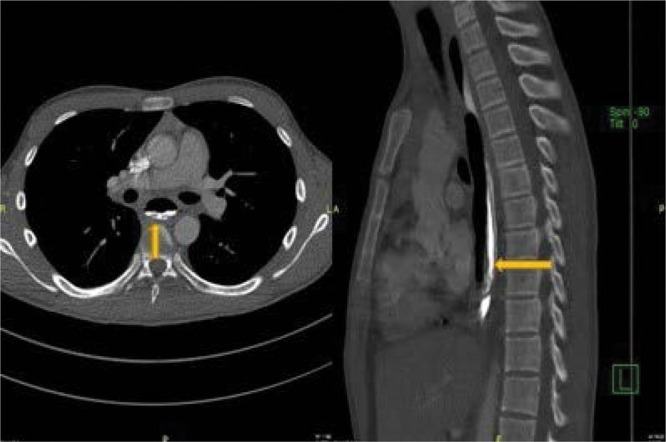
Computerized tomography. The retention of oral contrast in the false lumen and posterior of the true lumen without leakage (yellow arrow).

**FIGURE 2.**
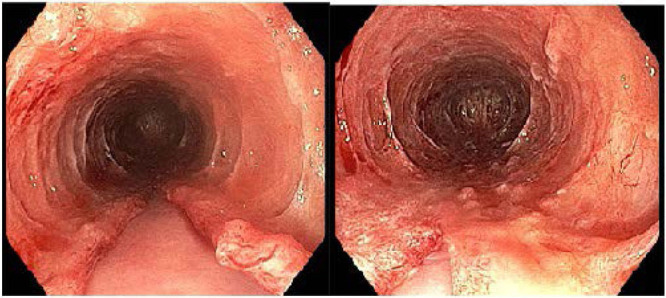
Upper endoscopy showing trachealization of mucosa and mucosal submucosal dissection.

A 14-year-old male patient was admitted with sudden onset of fever, chest, and epigastric pain. His complaints deteriorated in 7 days. His medical history was significant for food refusal, cough, and runny nose since early childhood when the patient had been evaluated for food allergy and gastroesophageal reflux disease (GERD). He responded well to treatment including ranitidine and lansoprazole (proton pump inhibitor [PPI]), in addition to dietary elimination of milk and egg. Since he was symptom-free, his family did not seek any further medical care. At current admission, physical examination was unremarkable. His laboratory workup were normal, except for an elevated level of C-reactive protein. Chest X ray was normal. Computerized tomography was performed revealing distal intramucosal dissection apparently immediately out of the wall in the middle and lower esophageal tract without leakage (Fig. 1). Total parenteral nutrition, pantoprazole, and piperacillin-tazobactam were initiated. Next, patient underwent upper endoscopy showing in esophagus crepe-paper-like mucosa, extensive ulceration with 5 cm mucosal defect starting 28 cm from the incisors ending in a cul-de-sac. The true lumen was visible followed distally by a pouch, measuring approximately 10 cm (Fig. 2). In light of these findings, EoE was considered and steroid 1 mg/kg/day was started with three-food elimination diet. However, biopsies showed ulceration and rare intraepithelial eosinophils in esophageal epithelium. Further examinations showed high serum immunoglobulin E levels (3427 U) and peripheral blood eosinophilia (450/mm^3^). Food allergy tests were unremarkable. The patient was discharged on PPI with oral viscous budesonide (OVB) on day 7. At follow-up 4 weeks after therapy, a repeat endoscopy showed an almost complete epithelialized laceration with no residual mucosal tears. Histopathological exam was inconclusive for EoE. OVB continued as 1 mg twice a day with PPI for 3 months. Thereafter, OVB was decreased to 1 mg/day. Due to his poor dietary compliance and depressiveness, his dietary restriction was also tapered. Symptoms of throat clearing and dysphagia recurred after meat and egg introduction. At 6 months, endoscopy showed loss of vascularity and exudative linear furrowing. Pathological examination revealed basal cell hyperplasia, eosinophilic microabscesses, and abundant eosinophil counts (100 eos/hpf), consistent with EoE. Three-food elimination diet was restarted with high dose OVB (2 mg/day) and continued for 3 months after that dose was tapered. He remained asymptomatic and compliant to maintenance treatment with OVB (0.5 mg/day). Follow-up endoscopy and histopathological examination was normal at 9 months and 1 year.

## DISCUSSION

Eosophageal intramural dissection is a rare clinical entity consisting out of the separation of mucosa and/or submucosa from deeper muscular layers with development of a false lumen as a consequence. Most cases are idiopathic, but EID may also occur after endoscopic interventions, ingesting sharp objects, and emetogenic in the presence of chronic inflammation and fibrosis like EoE (4). Uncontrolled remodeling reduces the elasticity and compliance of esophagus and may lead to EID, which can occur during endoscopic procedures and traction movements around the esophagogastric junction associated with nausea and vomiting. Prolonged symptoms and higher density of eosinophilic infiltration are risk factors for EID and rupture in EoE (5). Despite clinical and endoscopic findings which were compatible with EoE, the initial pathological examinations did not meet criteria for EoE. The lack of diagnostic eosinophilic infiltration may be related to advanced fibrosis, which showed correlation with duration of disease. Schoepfer et al (6) demonstrated that risk of developing esophageal strictures-fibrosis is significantly associated with the length of diagnostic delay, which is longest in young patients and gradually decreases with age. He had symptoms from early childhood. There was approximately 10 years between symptoms and diagnosis. His recurrent symptoms were considered as a part of GERD and asthma. Secondly, biopsy specimens may not be sufficient for assessing the esophageal subepithelium where esophageal remodeling occur. Eosinophils in EoE infiltrate not only the epithelial layers but also permeate throughout the esophageal wall, including the lamina propria, submucosa, and myenteric plexus (7). These clinical implications determine dysphagia and explain not only strictures and dysmotility but also the deep esophageal tears and perforations which result from vomiting.

Currently, guidelines on diagnosis and overall treatment of EoE do not provide any suggestions about the management of complicated cases, such as in EID. Conservative management is indicated when perforation is contained, conversely, in the suspicion of a severe perforation, management should be aggressive potentially considering surgery. The patient was treated with PPI, steroid, and elimination diet. He responded well and the epithelization of mucosal defect was achieved at first month. The short-term rapid histologic remission may be the consequence of combination therapy. Studies demonstrated significant improvement in esophageal subepithelial fibrosis with topical steroids in EoE. Elimination diet also reduces esophageal eosinophilia, however, fibrostenotic EoE is less likely to respond favorably than inflammatory EoE. Maintenance therapy and duration is also unclear for these patients. He received OVB for 1 year and stopped after 2 normal biopsies.

In conclusion, EID is rare but with serious consequences of esophageal remodeling which develop overtime in undiagnosed patients. Early diagnosis and institution of an effective treatment is paramount in preventing long-term sequelae of EoE. So, it should be considered in children with dysphagia and persistent GERD symptoms and early endoscopic evaluation should be undertaken to avoid diagnostic delay, especially in those with personal history of a significant atopic comorbidities.
